# Increased SBPase activity improves photosynthesis and grain yield in wheat grown in greenhouse conditions

**DOI:** 10.1098/rstb.2016.0384

**Published:** 2017-08-14

**Authors:** Steven M. Driever, Andrew J. Simkin, Saqer Alotaibi, Stuart J. Fisk, Pippa J. Madgwick, Caroline A. Sparks, Huw D. Jones, Tracy Lawson, Martin A. J. Parry, Christine A. Raines

**Affiliations:** 1 School of Biological Sciences, University of Essex, Wivenhoe Park, Colchester CO4 3SQ, UK; 2 Centre for Crop Systems Analysis, Wageningen University, 6700 AK, Wageningen, The Netherlands; 3 Rothamsted Research, West Common, Harpenden, Hertfordshire AL5 2JQ, UK; 4 Institute of Biological, Environmental & Rural Sciences, Aberystwyth University, Gogerddan, Aberystwyth SY23 3EE, UK; 5 Lancaster Environment Centre, Lancaster University, Lancaster, UK

**Keywords:** sedoheptulose-1,7-biphosphatase, Calvin–Benson cycle, transgenic, biomass, yield

## Abstract

To meet the growing demand for food, substantial improvements in yields are needed. This is particularly the case for wheat, where global yield has stagnated in recent years. Increasing photosynthesis has been identified as a primary target to achieve yield improvements. To increase leaf photosynthesis in wheat, the level of the Calvin–Benson cycle enzyme sedoheptulose-1,7-biphosphatase (SBPase) has been increased through transformation and expression of a *Brachypodium distachyon* SBPase gene construct. Transgenic lines with increased SBPase protein levels and activity were grown under greenhouse conditions and showed enhanced leaf photosynthesis and increased total biomass and dry seed yield. This showed the potential of improving yield potential by increasing leaf photosynthesis in a crop species such as wheat. The results are discussed with regard to future strategies for further improvement of photosynthesis in wheat.

This article is part of the themed issue ‘Enhancing photosynthesis in crop plants: targets for improvement’.

## 1. Introduction

To meet the future demands of an increasing world population for food and fuel, crop yields need to be improved substantially. During the latter half of the twentieth century, agricultural yields largely rose in line with demand, mainly due to advances through breeding and farming practices. However, wheat yield increases in many parts of the world have plateaued in recent years, while at the same time a predicted 70% increase in yield will be needed over the next four decades to feed the growing population [1,2]. It is unlikely that more land can be used for the production of food, stressing the need to improve crop yields that can be obtained from existing arable land. Moreover, these improvements will have to be realized in a changing climate, where [CO_2_] is expected to increase from the current level of 400–550 ppm in the next four decades [3,4].

To meet this challenge it is necessary to increase the yield potential of crops (*Y*_p_) which is the product of primary production over a growing season (*P*_n_) and harvest index (*η*) when grown under optimal conditions [5]. The harvest index, *η*, is the partitioning of biomass into harvestable product and is already close to its theoretical limit in most cereals [6–10]. *P*_n_ is the product of the sum of solar irradiance over the growing season (*S*_t_), the efficiency of canopy light interception (*ɛ*_i_) and the efficiency of conversion of captured light into biomass (*ɛ*_c_) divided by the energy content of the plant mass (*k*). Given that *S* is determined by the geographical location and *k* by the crop species, *Y*_p_ is predominantly determined by the efficiencies *ɛ*_i_ and ɛ_c._ Similar to *η*, *ɛ*_i_ has been improved substantially and is also near its theoretical maximum and is therefore not expected to greatly increase *Y*_p_, e.g. through breeding [11,12]. This leaves ɛ_c,_ which is not yet near its theoretical maximum of 4.6% [13] and there is therefore potential to improve this parameter in wheat [14] and in other crop species [11,12,15,16]. The primary targets for crop improvement would therefore be enhancement of photosynthesis per unit leaf area and optimization of canopy light distribution. Evidence for increased yield in response to CO_2_ enrichment has been shown consistently [17], providing compelling evidence that yields can be increased through the improvement of photosynthesis. For wheat, this is substantiated by the positive relationship between photosynthesis and biomass [18] and yield [19]. However, it should be noted that for CO_2_ enrichment studies of both wheat and other crops, measured yields in the field were lower than predicted [15,20,21]. This discrepancy indicates that, among potential other factors, the ratio of source (CO_2_ assimilation) to sink (demands of plant organs for assimilates) is also sub-optimal. In summary, there is evidence that improving photosynthesis can achieve significant enhancement of *Y*_p_ [14,16,22]; however, in the future it is likely that optimization of the source–sink ratio will be required in order to maximize the potential gains [7].

The Calvin–Benson cycle is the primary CO_2_ assimilation pathway and is co-limited by the maximum carboxylation efficiency of Rubisco (*V*_c,max_) and the regeneration of the substrate RuBP driven by photosynthetic electron transport (*J*_max_). Under current atmospheric [CO_2_] and saturating light intensity, CO_2_ assimilation operates at the transition between the Rubisco and the RuBP limited phases. However, CO_2_ assimilation will move towards being RuBP limited as a result of the predicted increase in atmospheric [CO_2_]. Both Rubisco and sedoheptulose-1,7-biphosphatase (SBPase) have been shown to exert control over flux of CO_2_ through the Calvin–Benson cycle. SBPase is the first step in the Calvin–Benson cycle that commits intermediates to the regeneration of RuBP through the dephosphorylation of sedoheptulose-1,7-biphosphate (SBP) to sedoheptulose-7-phosphate (S7P). The importance of SBPase in the control of the regenerative phase of the Calvin cycle has been shown by several studies; modelling and simulation studies have shown that SBPase exerts control over the rate of RuBP regeneration [23,24]. In studies that decreased SBPase levels in tobacco plants, reduced photosynthesis and growth were observed [25–27], with similar results found for rice [28]. Conversely, increases in the activity levels of SBPase led to increased photosynthesis and biomass in tobacco under controlled conditions [29] as well as in the field under elevated [CO_2_] [30]. In food crops such as tomato, increased SBPase activities resulted in similar increases in photosynthesis and growth, as well as improved chilling tolerance [31]. In rice, photosynthesis and growth were less affected by drought and heat stress in plants overexpressing SBPase, but no changes in biomass and yield were found [32,33]. This shows that the effects of increased SBPase activity on photosynthesis are positive, but effects on plant growth or yield may vary, particularly in important food crop species. Clearly, these results provide evidence to suggest that increasing the activity of SBPase has the potential to improve photosynthesis.

For one of the most important food crops in the world, the effect of increasing SBPase has not been investigated. Wheat provides more than 20% of calories consumed by the global population [34]. However, increases in wheat productivity (1.1% per year [35]) are below the predicted global demand in coming decades (1.7% per year [36]). Increasing photosynthesis in wheat is seen as one of the ways to meet this challenge, and increasing SBPase has been proposed as a means to achieve this [7]. Therefore, this study investigates whether *Y*_p_ in wheat can be realized through increases in SBPase. The effects of increased SBPase in wheat were studied through generation of transgenic wheat plants expressing a *Brachypodium distachyon* gene for SBPase, to elevate the total SBPase levels and activity. These plants were not only analysed for effects on photosynthetic capacity and growth, but also for their biomass and yield components under controlled conditions. The results are discussed in relation to the possibilities of increasing levels of SBPase for increasing *Y*_p_ in wheat.

## 2. Material and methods

### (a) Production and selection of transgenic plants

A functional *Brachypodium distachyon* SBPase genomic and cDNA hybrid sequence was generated and cloned into a modified pBract302 vector, inserted in between the rice tungro virus promoter (RTVP) and 35S terminator (electronic supplementary material, figure S1). The plasmid contained the *nptI* kanamycin resistance gene for selection of bacteria and the *bar* gene for phosphinothricin resistance under control of the *Zea mays* ubiquitin promoter for plant selection. This was co-transformed with pAHC20 which contains the *bar* gene under the control of the Ubiquitin1 promoter sequence + intron [37] due to the inefficient selection provided by the pBract302 *bar* gene. The recombinant plasmids were introduced into wheat cv. Cadenza by particle bombardment of wheat embryos, as described by Sparks and Jones [38,39]. From bombardments, 25 independent transgenic plants were identified by PCR, self-fertilized, and T_1_ and T_2_ plants were used for further selection of stably transformed plants. In parallel, particle bombardment was done using an empty vector to generate wild-type (WT) lines that had gone through the transformation process but had no foreign DNA inserted.

### (b) Growth conditions

Seeds of T_3_ and T_4_ generations were germinated and seedlings were grown in compost (Levington F2S, Fisons, Ipswich, UK) in a climate controlled room for three weeks (22°C, 12 h photoperiod). Selected seedlings were then transferred into 4 l pots (1 plant per pot), to a controlled environment greenhouse (25–32°C day/18°C night), with a 16 h photoperiod of natural irradiance (supplemented with high pressure sodium lamps, to a minimum light level of 175 µmol m^−2^ s^−1^ PAR). Plants of the T_3_ generation were grown in the greenhouse at the University of Essex (Colchester, UK) during the period of April to September (2014) at a constant density of 25 plants m^−2^. To reduce the effect of canopy-induced shading and increase the chance of tillering, plants of the T_4_ generation were grown in the same greenhouse from March to August (2016) at a lower initial density of 18.75 plant m^−2^ and from Zadoks stage 4.1 at a density of 15.6 plants m^−2^. A constant, high plant density (T_3_ plants) and a lower density (T_4_ plants) allowed assessment of plant growth with and without the influence of canopy-induced shading. All plants were regularly watered and moved to minimize spatial variation of growth conditions.

### (c) DNA extraction, RNA isolation and cDNA synthesis

A high throughput DNA extraction method was used on the first leaf of two week old seedlings. Leaf material (*ca* 0.1 g fresh weight) was collected in 1.2 ml micro-collection tubes in 96-tube racks (Starlab UK Ltd, Milton Keynes, UK) on dry ice and subsequently stored at −80°C. Frozen samples were dried overnight in a freeze dryer. To each tube, one stainless steel ball bearing was added, sealed well and ground in a Retch mill (TissueLyser, Qiagen, Manchester, UK) for 5 min at a frequency of 25 s^−1^. To the finely ground tissue, 600 µl of extraction buffer (0.1 M Tris-HCl, 0.05 M EDTA, 1.25% SDS, pre-heated to 65°C) was added per tube. Tubes were closed, mixed well and incubated for 30 min at 65°C. Tubes were cooled to below room temperature in the fridge for 15–30 min. then, 300 µl of cold 6 M ammonium acetate was added to each tube, closed, mixed well and incubated for 15 min at 4°C. Tubes were centrifuged for 15 min at 4°C at 13 000 g and 600 µl of supernatant added to new micro-collection tubes containing 360 µl of isopropanol and incubated for 5 min at room temperature. Tubes were centrifuged for 15 min at 13 000 ***g*** and the supernatant was discarded. 500 µl of 70% ethanol was added to the pellet, centrifuged for 15 min at 13 000 ***g*** and supernatant discarded. The pellet was resuspended in 400 µl distilled H_2_O and left overnight at 4°C. Tubes were centrifuged for 20 min at 13 000 ***g*** and 300 µl of supernatant was transferred and stored at −20°C until further use.

Total RNA was isolated from frozen leaf material (*ca* 0.1 g fresh weight; FW) ground in liquid nitrogen using Tri-reagent (Sigma T9429), modified from Hilario and Mackay [40] with the following additional steps: 30 µl of 3 M sodium acetate and 750 µl of ice-cold absolute ethanol were added and centrifuged at 13 000 ***g*** at 4°C for 15 min and the sample was then left on dry ice for 20 min. The RNA pellets were then washed with 75% ethanol and centrifuged for 5 min. Finally, the pellets were allowed to air-dry for 10 min before re-suspension in 50 µl of purified water and were stored at −80°C. The extracted RNA was quantified via absorbance measurement at 260 nm using a NanoDrop spectrophotometer (Nanodrop Products, Wilmington, USA). cDNA was synthesized from mRNA using SuperScript first strand cDNA synthesis (Invitrogen, UK) as specified by the manufacturer.

### (d) PCR and qPCR

Plants were screened for the presence of the construct from isolated DNA by PCR. Expression of the construct was determined from cDNA by qPCR and expressed relative to the gene expression of a stable reference gene for wheat (Ta2291 [41]). qPCR reactions were performed using SensiFast SYBR No-ROX mix (Bioline Reagents Ltd, London, UK) as specified by the manufacturer. Fold expression was determined according to Pfaffl [42]. Oligo sequences of primers used, with details for both PCR and qPCR are provided in the electronic supplementary material, table S1.

### (e) Determination of SBPase gene copy number

For analysis of the SBPase copy number of the inserted gene construct, *g-Count* technology was used to estimate copy number compared to a reference gene and to predict zygosity on leaf material from T_4_ plants of the transformed lines. Quantitative real time PCR analysis was used to estimate the numbers of transgene copies in individual wheat plants, similar to the approach taken for barley by Bartlett *et al.* [43]. An amplicon from the SBPase gene (with a FAM reporter) and an amplicon from a wheat internal positive control (IPC, with a VIC reporter) were amplified together in a multiplex reaction (15 min denaturation, then 40 cycles of 15 s 95°C and 60 s 60°C) in an ABI7900 real-time PCR machine. Fluorescence from the FAM and VIC fluorochromes was measured during each 60°C step, and the *C*_t_ values obtained. The difference between the *C*_t_ values for the SBPase gene and the IPC (the Δ*C*_t_) was used to allocate the assayed samples into groups with the same gene copy number. Analysis was carried out by iDNA Genetics Ltd (Norwich, UK).

### (f) Analysis of biomass and yield

Plant aboveground biomass was determined at full physiological maturity (Zadoks 9.1–9.2). For T_3_ plants, stems, leaves and ears (with seeds) were separated and counted, dried at 70°C (until a constant dry weight was reached) and weighed. For T_4_ plants, the whole plant was dried and weighed, without separation of stems and leaves. For all plants, ears were subsequently threshed and seeds were counted and weighed.

### (g) Protein extraction and western blot analysis for SBPase

Leaf samples (*ca* 0.2 g FW) were ground in liquid nitrogen, extracted and quantified essentially as described by Harrison, *et al.* [26]. Equal total protein of samples was loaded onto 12% (w/v) SDS-PAGE gels, separated and transferred onto a nitrocellulose membrane. Proteins were probed using antibodies raised against SBPase. The antibodies were raised using peptide immunization in rabbit, carried out by Cambridge Research Biochemicals (Billingham, UK). Proteins were detected using horseradish peroxidase conjugated to the secondary antibody and Pierce ECL chemiluminescence detection reagent (Thermo Scientific, Rockford, IL) and visualized using a FUSION FX chemiluminescence detection system (PEQLAB Ltd, Sarisbury Green, UK).

### (h) Total SBPase activity

Leaf SBPase activity was determined by phosphate release from SBP, as described by Harrison *et al.* [26] and Lefebvre *et al.* [29], with modifications. Leaf material (*ca* 5–6 cm^2^) was taken directly after measurement of photosynthesis, snap-frozen in liquid nitrogen and stored at −80°C. Leaf material was ground in liquid nitrogen to a fine powder after which 1.75 ml of extraction buffer (50 mM HEPES, pH 8.2; 5 mM MgCl_2_; 1 mM EDTA; 1 mM EGTA; 10% glycerol; 0.1% Triton X-100; 2 mM benzamidine; 2 mM aminocapronic acid; 0.5 mM phenylmethylsulfonylfluoride; 10 mM dithiothreitol) was added, mixed well and centrifuged for 3 min at 13 000 ***g*** at 4°C. 1 ml supernatant was purified through a pre-equilibrated desalting column (Illustra NAP-10, GE Healthcare Life Sciences, Little Chalfont, UK) and eluted with 1.5 ml of desalting buffer (extraction buffer excluding Triton X-100). The eluate was aliquoted, snap frozen in liquid nitrogen and stored at −80°C. For the assay, the reaction was started by adding 20 µl of extract to 66 µl of assay buffer (50 mM Tris, pH 8.2; 15 mM MgCl_2_; 1.5 mM EDTA; 10 mM dithiothreitol; 2 mM SBP) and incubated at 25°C for 10 min. The reaction was then cooled on ice and stopped by adding 50 µl of 1 M perchloric acid. Samples were centrifuged for 10 min at 13 000 ***g*** at 4°C and 30 µl of supernatant and 30 µl of phosphate standards (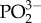, 0.125–8 nmol) were incubated in a 96 well microtitre plate for 30 min at room temperature with 300 µl of Biomol Green (Enzo Life Sciences Ltd, Exeter, UK). Absorbance of the resulting reaction was measured at 620 nm using a microplate reader (SpectroStar Omega, BMC Labtech, Aylesbury, UK).

### (i) *A*/*c*_i_ photosynthetic gas exchange measurements

Photosynthetic gas exchange measurements were performed on fully emerged flag leaves for the T_3_ generation plants, between flag leaf sheath extension and boot (sheath) swelling (Zadoks growth stages 4.1–4.5). The response of CO_2_ assimilation (*A*) to changes in intercellular [CO_2_] (*c*_i_) was measured in the middle of the flag leaf with a saturating irradiance of 2000 µmol photons m^−2^ s^−1^, using an open infrared gas exchange system and 6 cm^2^ leaf chamber with a blue–red LED light source (LI-6400-02B; LI-COR, Lincoln, NE). Leaves were clamped in the leaf chamber and complete sealing of the gaskets around the leaf was ensured to prevent possible diffusion leakage. For plants of the T_3_ generation, leaf temperature was maintained at 20 ± 1°C with a vapour pressure deficit of 0.9 kPa and an ambient [CO_2_] (*c*_a_) of 400 µmol mol^−1^. Subsequently, *c*_a_ was decreased to 300, 200, 100 and 50 µmol mol^−1^ before returning to the initial concentration. This was followed by an increase to 650, 900, 1200 and 1500 µmol mol^−1^. Readings were recorded when *A* had stabilized to the new conditions (after about 2 min). Similarly, for plants of the T_4_ generation, leaf temperature was maintained at 22 ± 1°C with a vapour pressure deficit of 1.3 kPa and *c*_a_ of 400 µmol mol^−1^. Subsequently, *c*_a_ was decreased to 300, 250, 150, 100 and 50 µmol mol^−1^ before returning to the initial concentration. This was followed by an increase to 550, 700, 900, 1100, 1300 and 1500 µmol mol^−1^. Readings were recorded when *A* had stabilized to the new conditions (after about 2 min).

### (j) Model fitting of *A*/*c*_i_ response

Model fitting for *A*/*c*_i_ response curves was after the method of Dubois *et al.* [44] using the Farquhar *et al.* [45] model. Parameters estimated were: the maximum velocity of Rubisco for carboxylation (*V*_c,max_) and the maximum rate of electron transport demand for RuBP regeneration (*J*_max_).

All calculations were done in the R environment, v. 3.2.1 [46].

## 3. Results

### (a) Production and selection of transformants

**Figure 1.**
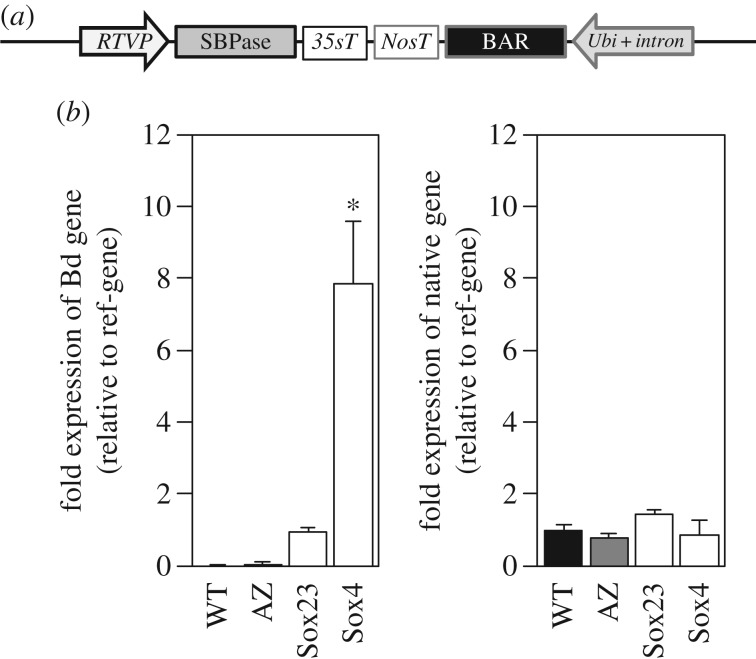
(*a*) Schematic representation of the construct used for transformation (see also electronic supplementary material, figure S1). (*b*) Relative gene expression of the *Brachypodium* SBPase construct (Bd gene) and the native SBPase gene T_3_ lines of wild-type (WT), azygous control (AZ), lines Sox23 and Sox4. Measurements were done on fully developed flag leaves (Zadoks 4.1) of greenhouse grown plants. Means and standard error (*n* = 5), asterisks indicate significant difference from WT (*p* < 0.05).

**Figure 2.**
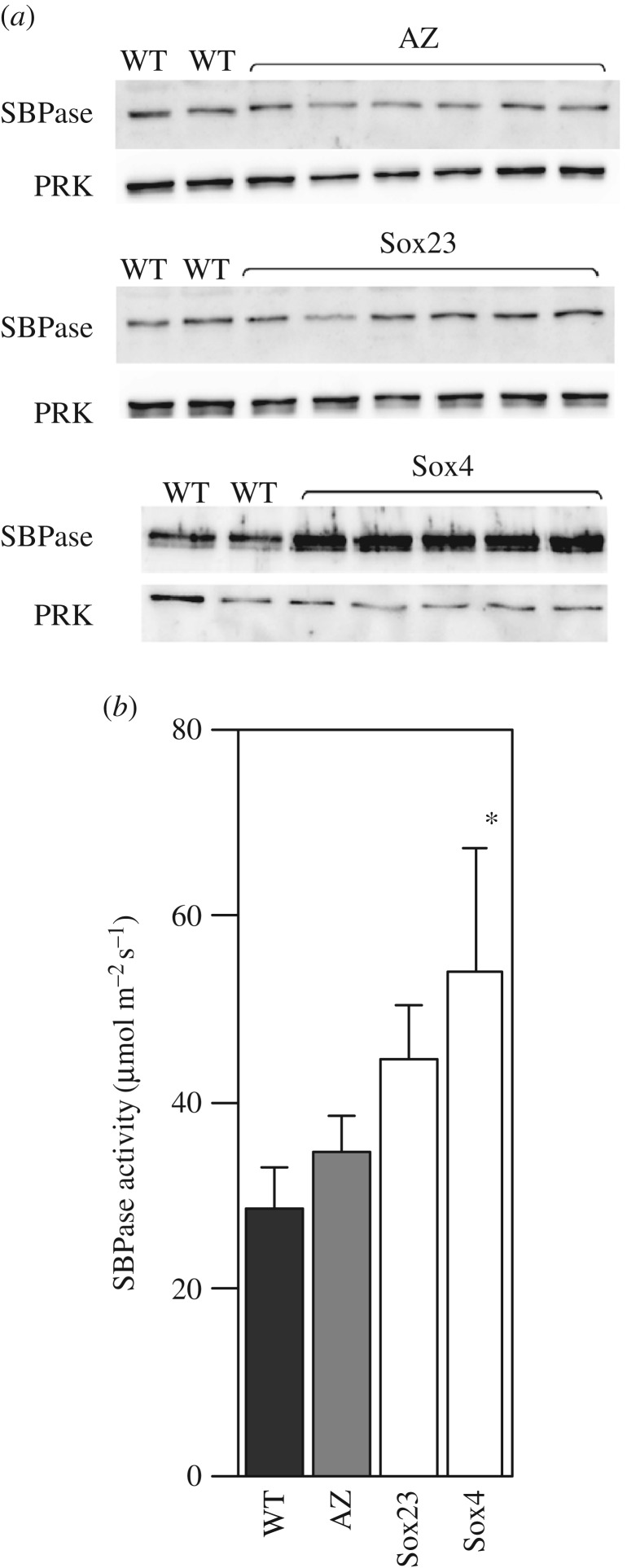
(*a*) Immunoblot analysis of leaf protein extracts from the Sox T_3_ lines (see figure 1). The same samples were used to probe for PRK. (*b*) SBPase activity of T_3_ lines. Measurements were done on fully developed flag leaves (Zadoks 4.1) of greenhouse grown plants. Means and standard error (*n* = 5), asterisks indicate significant difference from WT (*p* < 0.05).

A chimeric gene construct consisting of a genomic- and cDNA SBPase hybrid sequence from *Brachypodium distachyon* was cloned into a modified vector (pBract302), containing an RTVP and a 35S CaMV terminator (figure 1*a* and electronic supplementary material, figure S1). The *nptI* kanamycin resistance gene in the vector was used for selection of bacteria. Although the pBract302 vector also carried a *bar* gene for phosphinothricin resistance, under the control of the *Zea mays* ubiquitin promoter plus first intron (*Ubi-1*)), it was known to be inefficient and consequently an additional plasmid pAHC20 [37] was used to introduce the *bar* gene for selection of transformed plants. The recombinant plasmids were introduced into *Triticum aestivum* cv. Cadenza by particle bombardment of wheat embryos, as described by Sparks and Jones [38,39]. This generated 25 independent primary transgenic plants (T_0_) identified by PCR analysis for both the SBPase and *bar* genes; these plants were allowed to self-fertilize and the resulting T_1_ seed collected. The subsequent T_1_ and T_2_ generation plants were grown under controlled conditions in a greenhouse and to confirm the presence of the construct, genomic DNA was isolated and screened for the gene of interest and *bar* gene using PCR. Expression of the introduced SBPase gene as well as the native SBPase gene in the flag leaves was determined using qPCR and, based on these results, two contrasting lines were chosen (Sox23 and Sox4) to study the effect of increased SBPase activity on wheat. The relative transcript levels of the SBPase transgene in the T_3_ plants of the Sox23 and Sox4 lines (compared to a reference gene *Ta2991* [41]), were shown to be increased relative to wild-type and azygous plants (figure 1*b*), without a significant effect on the expression of the native SBPase gene (figure 1*b*). In flag leaves of these T_3_ plants, protein levels of SBPase were also found to be increased in comparison to wild-type (WT), while PRK protein levels remained constant (figure 2*a*). Transgene expression and SBPase protein levels verified the functionality of the introduced construct into wheat, resulting in plants differing in SBPase content. Plants of the two T_3_ lines with different SBPase protein levels were grown under greenhouse conditions and total SBPase activity of the flag leaf was determined. Increased SBPase activity was found in both Sox23 and Sox4 plants, with the latter having the highest activity, transcript and protein levels (figure 2*b*).

### (b) The effect of increased SBPase activity on CO_2_ assimilation, plant biomass and seed production

**Figure 3.**
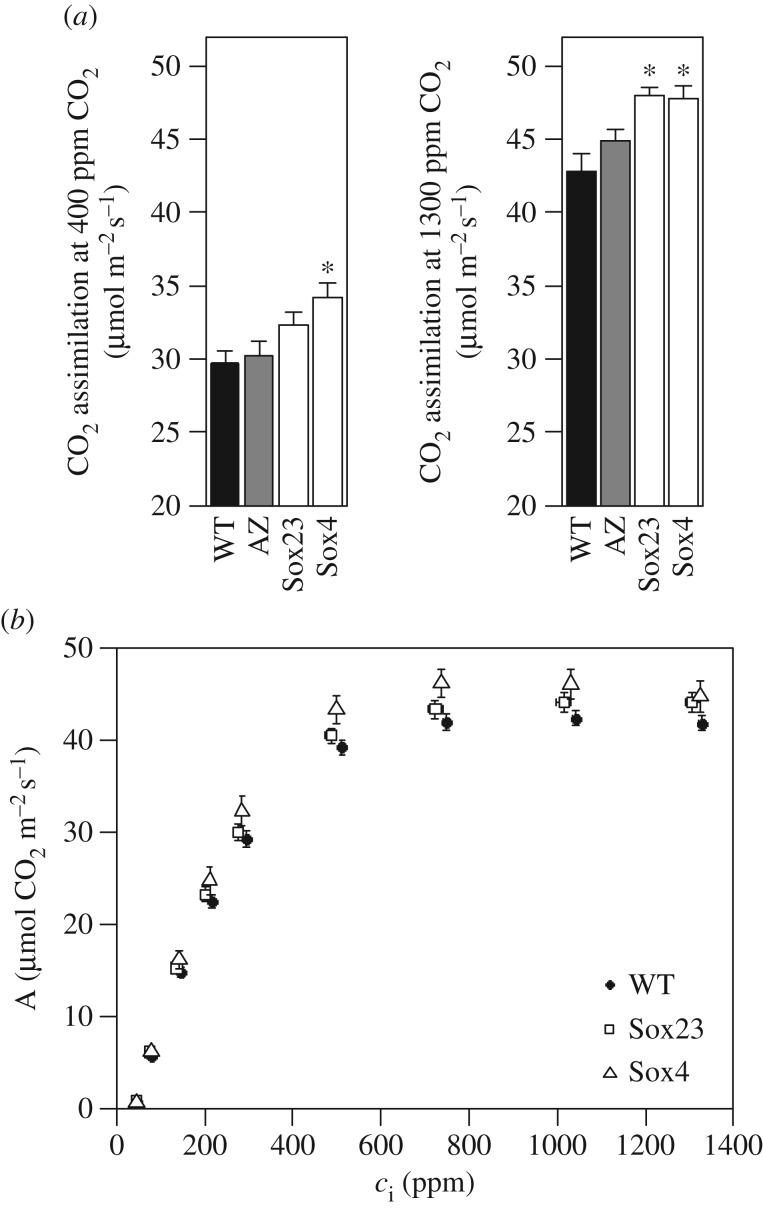
(*a*) CO_2_ assimilation rates of Sox T_3_ lines at ambient [CO_2_] (400 ppm) and saturating [CO_2_] (1300 ppm). Means and standard error (*n* = 5). Asterisks indicate significant differences from WT (*p* < 0.05). (*b*) Response curves of T_3_ lines of CO_2_ assimilation rate (*A*) to changes in intercellular [CO_2_] (*c*_i_). Measurements were done on fully developed flag leaves (Zadoks 4.1) of greenhouse grown plants. Means and standard error (*n* = 5).

T_3_ SBPase transgenic and WT plants were grown in a controlled environment greenhouse (25–32°C day/18°C night) at a plant density of 25 plants m^−2^ from April to September, with a 16 h photoperiod of natural irradiance and supplemented with high pressure sodium lamps (minimum light level of 175 µmol m^−2^ s^−1^ PAR). At Zadoks stage 4.1, CO_2_ assimilation rates of the flag leaves were determined at both ambient and saturating [CO_2_] (400 and 1300 ppm [CO_2_], respectively, figure 3*a*). The Sox4 line, which had the higher SBPase activity, had higher rates of CO_2_ assimilation under both conditions, but in Sox23 CO_2_ assimilation was only significantly higher than WT at saturating [CO_2_] (figure 3*a*). The observed CO_2_ assimilation rates of the different lines had a positive, significant linear relationship with SBPase activity at both [CO_2_] values (electronic supplementary material, figure S2*a*,*b*). Further analysis of the photosynthetic rates of the flag leaves of these plants was carried out by determining the response of CO_2_ assimilation (*A*) to changes in intercellular CO_2_ concentration (*c*_i_). Both of the transgenic lines, Sox23 and Sox4, had a significantly different response of *A* to that of WT, particularly at high *c*_i_ (figure 3*b*.). However, although the average values obtained for *V*_c,max_ and *J*_max_ (table 1) were higher than for WT in both Sox 23 and Sox 4, these differences were not significant, with exception of *V*_c,max_ in the Sox4 line.

**Table 1.** *V*_c,max_ and *J*_max_ of the response of CO_2_ assimilation (*A*) to intercellular CO_2_ concentration for T_3_ plants of different lines. Parameters were fitted to the model of Farquhar *et al.* [45] according to Dubois *et al*. [44]. Means and standard error (*n* = 5), asterisks indicate significant difference from WT (*p* < 0.05). Mean and standard error (*n* = 4 or more).

line	*V*_c,max_	*J*_max_
WT	103 (2.8)	192 (3.7)
AZ	111 (4.6)	201 (6.5)
Sox23	109 (2.0)	195 (4.4)
Sox4	117 (5.1)*	210 (6.7)

*p < 0.05.

**Figure 4.**
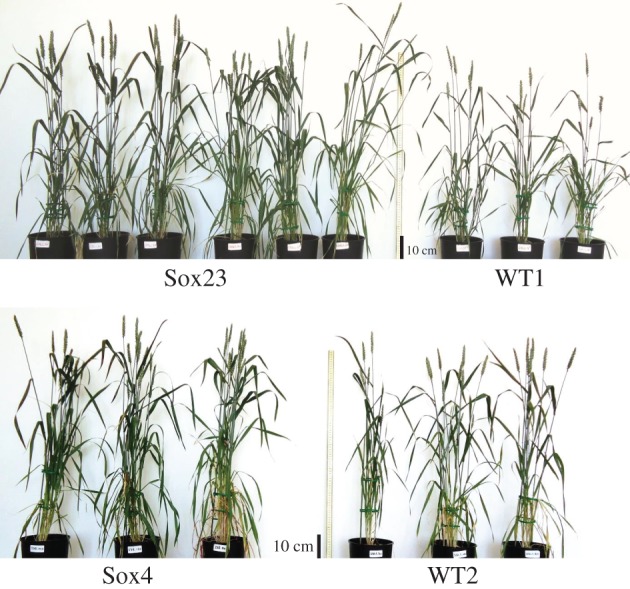
T_3_ plants (Zadoks stage 6.9) from Sox lines compared to wild-type plants (WT). Plants were grown under natural light, with supplementary lighting (minimum of 175 µmol m^−2^ s^−1^ PAR), in an environmentally controlled greenhouse in the period April–July 2014. Scale bar indicates 10 cm.

**Figure 5.**
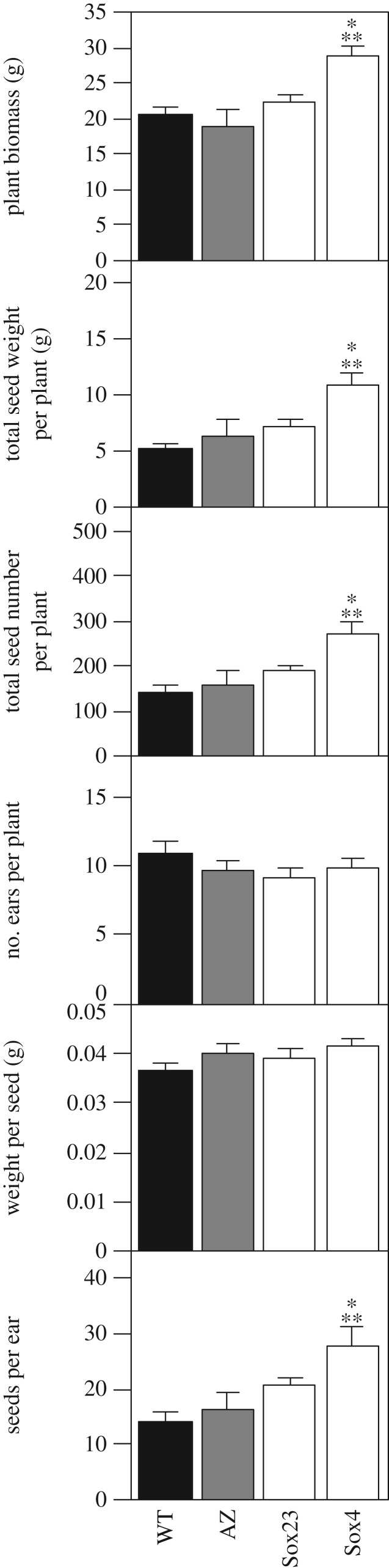
Biomass of T_3_ plants grown to full physiological maturity (Zadoks 9.1–9.2) with different SBPase activity; whole plant biomass, total dry seed weight per plant, total number of seeds per plant, number of ears per plant, average weight per seed, number of seeds per ear. Means and standard error (*n* = 4 or more). Asterisks (*) indicates significant difference from WT (*p* < 0.05), asterisks (**) indicates significant difference from AZ (*p* < 0.05).

Comparison of plants at Zadoks stage 6.9 with increased SBPase activity showed that Sox4 and Sox23 were visibly taller and produced a more dense leaf canopy, compared to WT plants (figure 4). The WT plants shown are representative of the range of differences in growth between two different transformation control groups (WT), generated in separate bombardment events. The vegetative biomass and total seed weight of WT and the transgenic Sox lines (T_3_) were determined at full maturity (Zadoks 9.1–9.2). This analysis showed that the total biomass and the seed weight per plant at maturity was *ca* 40% higher in the Sox4 plants (figure 5) and the means of the different lines had a positive, significant linear relation with SBPase activity (electronic supplementary material, figure S2*c*,*d*). The allocation of biomass to leaves was not significantly different between the Sox lines and WT, but the stem fraction was lower in line Sox4 and in this line the percentage allocated to ears was increased by 10% (electronic supplementary material, figure S3). The significant increase in the total seed weight in Sox4 was the result of an increase in the total number of seeds per plant as neither the number of ears nor the weight per seed differed significantly between the Sox lines and WT (figure 5). This led to a significantly higher harvest index *η* = 0.38 (0.02) for Sox4 compared to *η* = 0.25 (0.02) for WT (mean and standard error, *p* = 0.006).

### (c) Increased plant biomass and seed production was consistent across generations and growing conditions

To further explore the impact of increase SBPase activity on the biomass of wheat and given variation between individual T_3_ plants within lines, particularly in Sox4, the next generation of plants (T_4_) were produced. Prior to physiological analysis of these T_4_ progeny, the gene copy number was determined. Sox23 plants were found consistently to have six extra copies (12 in homozygous, table 2); in contrast, Sox4 was shown to have a segregation of copy numbers. This comprised plants with one copy (two in homozygous, table 2) and two functional copies (four in homozygous, table 2). Following this analysis, in all future experiments Sox4 was divided into two groups; plants with two gene copies (Sox42) or four gene copies (Sox44). It is noteworthy that the highest SBPase activities were found in the lines with the lowest copy numbers (Sox4, or Sox42 and Sox44) and lower SBPase activity in the line with high copy numbers (Sox23).

**Table 2.** Gene copy number of inserted construct for T_4_ plants.

line	gene copy number
WT	0
Sox23	6 (12 homozygous)
Sox42	1 (2 homozygous)
Sox44	2 (4 homozygous)

**Figure 6.**
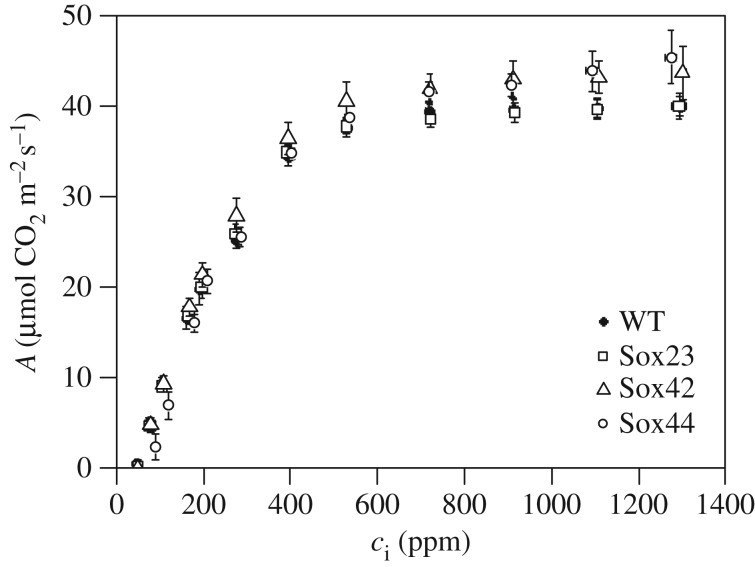
Response curves of T_4_ lines of CO_2_ assimilation rate (*A*) to changes in intercellular [CO_2_] (*c*_i_). Measurements were done on fully developed flag leaves (Zadoks 4.1) of greenhouse grown plants (May–August 2016). Means and standard error (*n* = 4).

In a second experiment T_4_ plants were grown in a controlled environment greenhouse (25–32°C day/18°C night), with a 16 h photoperiod of natural irradiance and supplemented with high pressure sodium lamps to a minimum light level of 175 µmol m^−2^ s^−1^ PAR. In this case plants were grown at a lower density (16–18 plants m^−2^) to increase the chance of tillering. The *A*/*c*_i_ response measured on flag leaves of T_4_ plants showed that lines Sox42 and Sox44 had a significantly different response than line Sox23 and WT (figure 6). Although no significant differences were observed in *V*_c,max_ between these lines and WT (table 3), *J*_max_ was increased in Sox42 and was significantly higher than WT in line Sox44 (table 3).

**Table 3.** *V*_c,max_ and *J*_max_ of the response of CO_2_ assimilation (*A*) to intercellular CO_2_ concentration for T_4_ plants of different lines. Parameters were fitted to the model of Farquhar *et al.* [45] according to Dubois *et al.* [44]. Means and standard error (*n* = 4), asterisks indicate significant difference from WT (*p* < 0.05). Mean and standard error (*n* = 3 or more).

line	*V*_c,max_	*J*_max_
WT	84 (2.6)	178 (4.0)
Sox23	92 (3.6)	184 (7.6)
Sox42	97 (8.5)	202 (17.7)
Sox44	101 (7.1)	222 (22.1)*

*p < 0.05.

**Figure 7.**
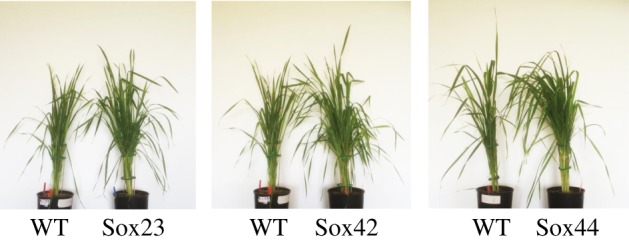
T_4_ plants from lines Sox23, Sox42 and Sox44 in comparison to wild-type plants (WT). Plants were grown under natural light, with supplementary lighting (minimum of 175 µmol m^−2^ s^−1^ PAR), in an environmentally controlled greenhouse in the period May–August 2016.

**Figure 8.**
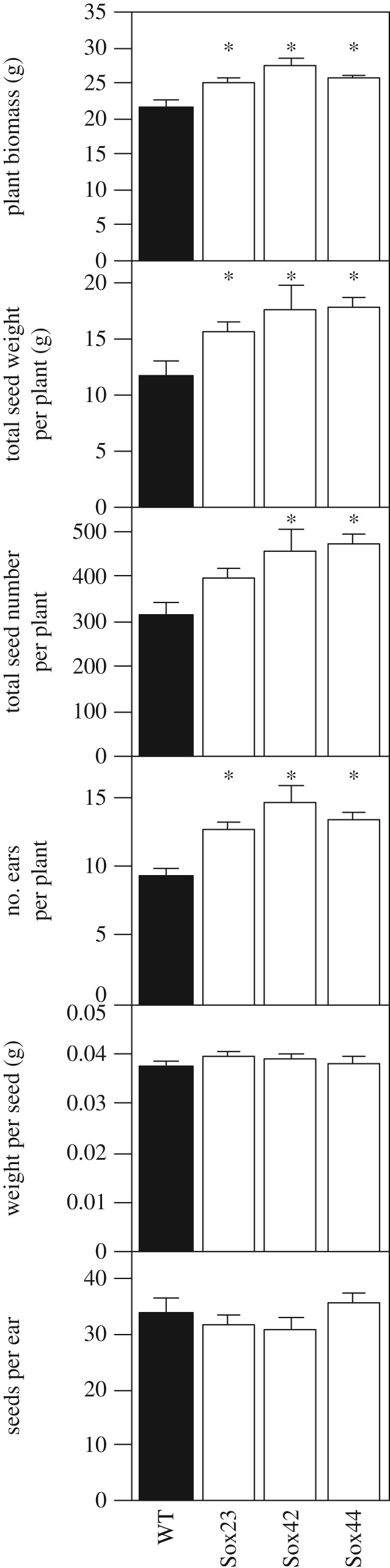
Biomass of T_4_ plants grown to full physiological maturity (Zadoks 9.1–9.2) with different SBPase activity; whole plant biomass, total dry seed weight per plant, total number of seeds per plant, number of ears per plant, average weight per seed, number of seeds per ear. Means and standard error (*n* = 5 or more). Asterisks (*) indicates significant difference from WT (*p* < 0.05).

Similarly to T_3_ generation plants, the T_4_ lines with increased SBPase activity were visibly taller with more foliage (figure 7). Furthermore, the vegetative biomass and the total seed weight of Sox23, Sox42 and Sox44 plants were increased significantly when compared to WT (figure 8). For both Sox42 and Sox44, the total number of seeds was increase significantly compared to WT plants; while a small increase was also observed in Sox23, this was not significant (*p* = 0.13, figure 8). The number of ears was also significantly greater for the Sox23, Sox42 and Sox44 plants compared to WT (figure 8).

## 4. Discussion

In this study we have examined the effects of increased SBPase protein levels and activity on photosynthesis and yield by expressing a *Brachypodium* SBPase gene-construct in wheat plants. It is shown that for an important crop such as wheat, increases in SBPase activity consistently resulted in an increase in leaf CO_2_ assimilation rate, particularly when measured under high [CO_2_]. Notably, the greatest increases in SBPase activity resulted not only in an increase in total biomass but also an increase in total seed weight (30–40% higher than WT). Two generations (T_3_ and T_4_) of transgenic SBPase overexpression wheat plants were grown under two different growth regimes: one grown in conditions which limited the chance of tillering (at high plant density) and the second in conditions that increased chances of tillering (at lower plant density). The results obtained were consistent between the two experiments. Total seed weight was found to be increased in both experiments in the plants with the highest SBPase activity. This was achieved either through a higher number of seeds being formed per ear (fewer tillers, at high plant density), or a larger number of ears being produced per plant (more tillers, at lower plant density). These results indicate that the positive effect of increased SBPase activity can be achieved at different plant densities. It also supports the contention that increasing SBPase activity in wheat has a positive effect on leaf photosynthetic capacity and, at least under controlled conditions, can lead to an increased *Y*_p_.

Higher CO_2_ assimilation rates were observed in the wheat plants displaying an increased SBPase activity. This is in agreement with effects of expression of either SBPase or the cyanobacterial SBPase/FBPase bifunctional enzymes that stimulated photosynthetic carbon assimilation and production of end products and/or biomass in other species [29,31,47–49]. The effects of increased SBPase activity in wheat plants on *J*_max_ and *V*_c,max_ were relatively small when compared to those observed for tobacco [29] and were only significantly different from the WT values in lines Sox4 (T_3_, for *V*_c,max_) and Sox44 (T_4_, for *J*_max_). As SBPase is involved in the regeneration of RuBP, a change in *J*_max_ would have been expected in this study. However, the effect of increased SBPase activity on this parameter can vary depending on growth conditions [16], which may explain the observed differences in either *V*_c,max_ or *J*_max_ between the two experiments. Nevertheless, we observed increased CO_2_ assimilation rates under light saturated conditions at both ambient and saturating [CO_2_] in plants that related positively and significantly with increased SBPase activity. Furthermore, CO_2_ assimilation rates for these plants were highest at saturating [CO_2_], which is in keeping with the role of SBPase in the regeneration of RuBP. Under future predicted atmospheric [CO_2_] the share of control over CO_2_ assimilation will move towards regeneration of RuBP and it is likely that the role of SBPase will become even more important under these conditions. Support for this comes from modelling of photosynthetic carbon metabolism by Zhu *et al.* [24] that identified a current underinvestment in SBPase levels. It was proposed that relatively large changes in SBPase levels should be made to achieve improved photosynthetic carbon metabolism. This suggests that there may be even more scope to increase further the biomass and seed yield of wheat and other crop species through introduction of higher levels of SBPase. Our results for wheat, in this current study, are consistent with this idea as the most significant positive effects were observed in the plants with the highest SBPase activity.

Further stimulation of photosynthesis may be obtained in wheat by the introduction of additional genes encoding photosynthesis proteins, similar to that shown recently for tobacco and *Arabidopsis* [48,49]. In this work further increases in biomass were obtained through the simultaneous over-expression of SBPase and fructose 1,6-bisphosphonate aldolase together with the glycine decarboxylase H subunit or the algal ictB protein [48,49]. In addition to stimulating photosynthesis a reduction of the Rubisco content may be desirable as this protein currently constitutes up to 50% of leaf N. This approach would have the potential to allow an N saving that may be reinvested in other proteins, e.g. SBPase (currently 1% of leaf N). However, this would only be useful if the reduction in Rubisco did not lead to a decrease in either photosynthesis or yield.

Previously a number of studies using model species showed that increased SBPase activity enhanced growth and photosynthesis under both controlled [29] and field conditions (tobacco) [30]. Increased SBPase activity may also be beneficial under elevated [CO_2_] and has also been shown to increase tolerance to salt and low and high temperatures [31–33]. This combination may prove beneficial under future climate conditions. To fully assess the potential of increased SBPase activity and improved photosynthesis for *Y*_p_ in wheat under future climates, the next step would be to assess effects under ambient and elevated [CO_2_] and increased temperature in the field. The results of the current study provide a clear demonstration that photosynthesis can be improved by manipulation of the enzymes of the Calvin–Benson cycle and that this can be applied in relevant crops such as wheat [14,22]. This greenhouse study forms the first step in translating research on improving photosynthesis in model species to application in important crops. The next stage of this work will be to undertake studies with these transgenic SBPase wheat plants under field conditions.

## Supplementary Material

Supplementary figures and table

## Supplementary Material

Data file 1

## Supplementary Material

Data file 2

## Supplementary Material

Data file 3

## Supplementary Material

Data file 4

## Supplementary Material

Data file 5

## Data accessibility

The datasets supporting this article have been uploaded as part of the electronic supplementary material.
