# Supplementary material for: Increased SBPase activity improves photosynthesis and grain yield in wheat grown in greenhouse conditions
Source: Philos Trans R Soc Lond B Biol Sci. 2017 Aug 14;372(1730):20160384. doi: 10.1098/rstb.2016.0384 (PMC5566882; doi:10.1098/rstb.2016.0384)
Supplement: Supplementary figures and table [file rstb20160384supp1.docx]

*Supplementary*

1 GCACCACATC AATAGAAGAT GAATGGATCC ATCGTATGGA CAATGCTTAA AGAAGCTTTA TCAAAAGCAA CTTTAAGTAC GAATCAATAA AGAAGGACCA

101 GAAGATATAA AGCGGGAACA TCTTCACATG CTACCACATG GCTAGCATCT TTACTTTAGC ATCTCTATTA TTGTAAGAGT GTGTAATGAC CAGTGTGCCC

201 CTGGACTCCA GTATATAAGG AGCACCAGAG TAGTGTAATA GATCATCGAT CAAGCAAGCG AGAGCTCAAA CTTCTAAGAG AGCAAGAGAG GAGCTTATCC

301 TCTCAACCCC TCTTCAAGAA GCAAAGGCGA TCAATGGCTC AGGTCAGTGA GTAGTCGTCT TTAAGGTTCC TCTAGGAACC TCTGTGTAAT ATGTTTGTAC

401 TTATGATTGT ATTTCTCAAA ACCTATCCGC TGCATGAATA AAGCTCTTAT ATTTATCCTA CACCCTTGTG ATAAAACATG AAGTCATACT CGTTCTAAGA

501 TTAAGTGTTC TAAAAATACT TCGAAAGAAT CACATTCTAT AGAGTCTGAT GACTCAATTC CACTATACAG GGACAAGCTT CCCGGACAGC AATGGAGACC

601 GTCGCGGCGT CGGGATACGC CCGCGGGGCC GCCACGCGCT CCCCCGCCTG CTGCGCCGCC ATGTCCTTCT CGCAGTCCTA CAGGCCCAAG GTGTGTGTGA

701 ATTTTGCAAA TGTAGGCGCG CCAAACTTTA TCTTTTTGGT TGATGCCTGA CGGGTAATGT CGGCGCGCTG CAGGCTGCCA GGCCGCCGAC CACGTTCTAC

801 GGCGAGTCGG TGCGGGCGAA CACGGCGAGG ACGCTCCCGG GGAGGCAGTC CAAGGCGGCG AGCCGCGCCG CGCTCACCAC ACGGTGCGCG ATCGGGGACA

901 GCCTGGTGAG TGTAGTGTAG ATAATCAGCC AATCAGCGTG CAGTGCCGAA CAAACTGTGC ATCGGTGCAG CCGTGAAAGT GCTTACGGTA CGCCGTGTTG

1001 TGGTGTGGTG CCAGGAGGAG TTCCTGACCA AGGCGACGCC GGACAAGAAC CTCATCAGGC TGCTGATCTG CATGGGGGAG GCGATGAGGA CGATCGCGTT

1101 CAAGGTCAGG ACGGCGTCCT GCGGCGGCAC GGCCTGCGTC AACTCCTTCG GAGACGAGCA GCTCGCCGTC GACATGCTCG CCGACAAGCT CCTCTTCGAG

1201 GCGTTGGAGT ACTCCCATGT CTGCAAGTAT GCGTGCTCTG AGGAAGTCCC TGAGCTGCAA GACATGGGTG GCCCAGTCGA CGGCGGTTTC AGTGTTGCGT

1301 TCGATCCCCT TGACGGTTCC AGCATTGTCG ACACAAACTT CACTGTGGGA ACCATCTTTG GTGTTTGGCC CGGTGACAAG CTGACGGGCG TCACCGGTGG

1401 AGACCAGGTT GCTGCTGCCA TGGGAATCTA CGGCCCTCGC ACCACTTTCG TAGTGGCCCT CAAGGACTGC CCTGGAACTC ATGAATTCCT TCTTCTTGAC

1501 GAAGGTAAAT GGCAGCATGT CAAGGACACC ACGACCATCG GAGAAGGGAA GATGTTCTCT CCTGGTAATC TGAGGGCCAC ATTTGACAAC CCTGATTATG

1601 ACAAGCTTGT CAACTACTAT GTCAAGGAGA AGTACACATT GCGCTATACC GGAGGAATGG TCCCTGATGT CAACCAGATC ATCGTCAAGG AGAAGGGCAT

1701 CTTCACCAAC GTGACGTCTC CCACGGCCAA GGCCAAGCTG CGGCTCCTGT TCGAGGTGGC CCCGCTGGGC TTCTTGATAG AGAAAGCCGG CGGGCACAGC

1801 AGCGACGGCA AGCAGTCGGT ACTGGACAAG GTGATCACGG TGCTCGACGA GCGGACCCAG GTGGCCTACG GGTCCAAGAA CGAGATCATC CGGTTCGAGG

1901 AGACTCTCTA CGGCTCGTCG AGGCTCGCGG CTGGCGCCAC CGTCGGCGCA ACCGTCTAAT CGATCGCTGA AATCACCAGT CTCTCTCTAC AAATCTATCT

2001 CTCTCTATTT TCTCCATAAA TAATGTGTGA GTAGTTTCCC GATAAGGGAA ATTAGGGTTC TTATAGGGTT TCGCTCATGT GTTGAGCATA TAAGAAACCC

2101 TTAGTATGTA TTTGTATTTG TAAAATACTT CTATCAATAA AATTTCTAAT TCCTAAAACC AAAATCCAGT ACTAAAATCC AGATCCCATC GCTGAAATCA

2201 CCAGTCTCTC TCTACAAATC TATCTCTCTC TATTTTCTCC ATAAATAATG TGTGAGTAGT TTCCCGATAA GGGAAATTAG GGTTCTTATA GGGTTTCGCT

2301 CATGTGTTGA GCATATAAGA AACCCTTAGT ATGTATTTGT ATTTGTAAAA TACTTCTATC AATAAAATTT CTAATTCCTA AAACCAAAAT CCAGTACTAA

2401 AATCCAGATC CCATCGCTGA AATCACCAGT CTCTCTCTAC AAATCTATCT CTCTCTATTT TCTCCATAAA TAATGTGTGA GTAGTTTCCC GATAAGGGAA

2501 ATTAGGGTTC TTATAGGGTT TCGCTCATGT GTTGAGCATA TAGAAACCCT TAGTATGTAT TTGTATTTGT AAAATACTTC TATCATAAAT TTCTAATTCC

2601 TAAAACCAAA TCCAGTACTA AATCCAGATC CCATCAGCTT ATCGATACCG TCGACTCGAG GGGG

RTVP SBPase (genomic/cDNA hybrid) 35S terminator (before this is T DNA, bases 1757-1928 from pGreen II; not on the map)

Suppl. Fig. 1. Sequence of the SBPase cassette, inserted into pGreenII plasmid. The SBPase cassette was inserted into the transformation vector pBract (backbone + Ubi::bar::nos cassette).


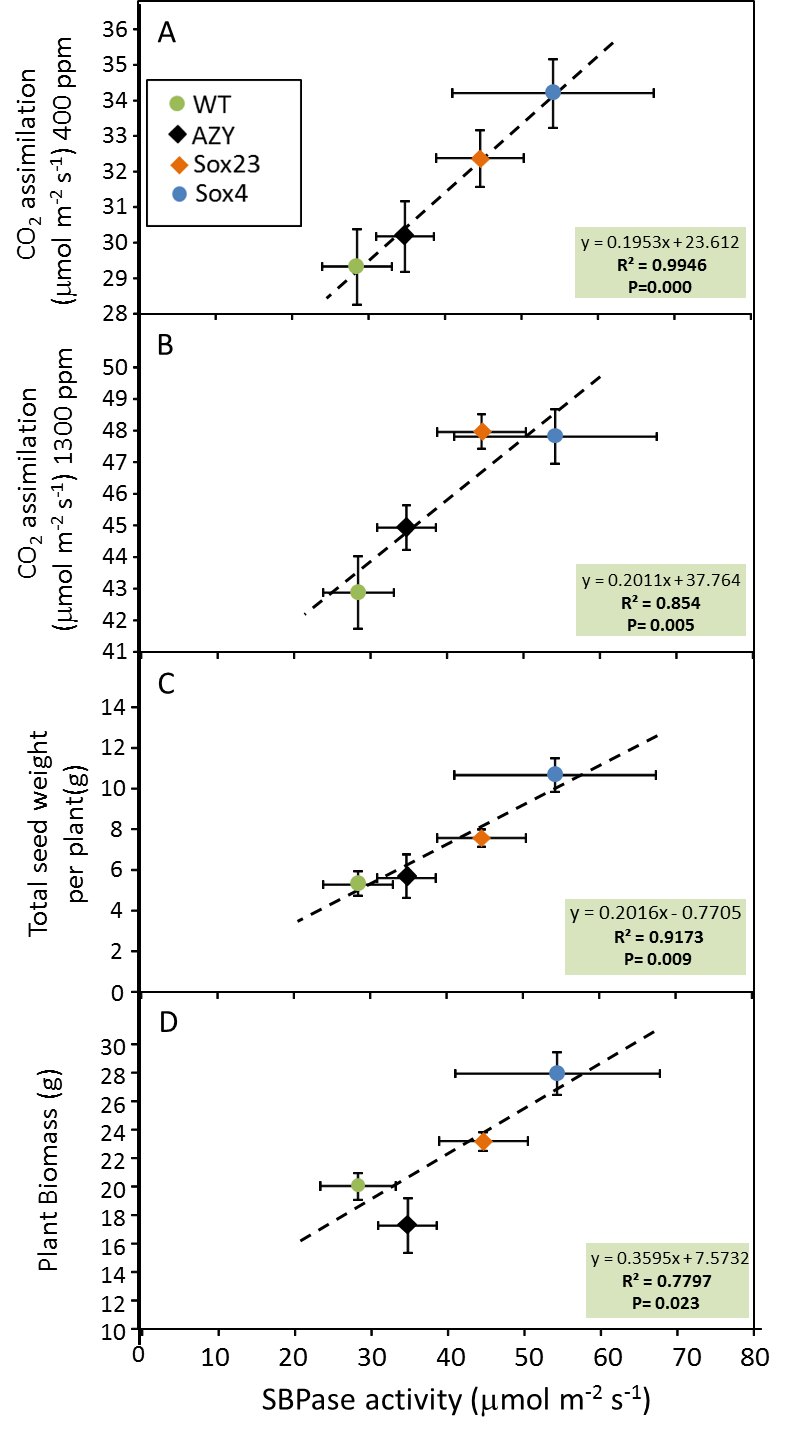


Suppl. Fig. 2. Relationship between leaf SBPase activity and (A) CO_2_ assimilation rate at ambient (400 ppm), (B) saturating [CO2] (1300 ppm), (C) Seed yield (g) and (D) total plant biomass (g) of T3 generation plants. Significant test for linear regression (P value) are shown for each lines.

Suppl. Fig. 3. Partitioning of biomass of T_3_ plants, grown to full physiological maturity (Zadok 9.1-9.2) with different SBPase activity; fraction of biomass to ears, fraction of biomass to leaves and fraction of biomass to stems. Means and standard error (n=4 or more). * indicates significant difference from WT (P<0.05).

*Suppl. Table 1. Oligo sequences used for PCR and qPCR*

| Oligo sequence:  Forward/  Reverse | Target | PCR/  qPCR | Optimized primer concentration [μM] | Amplicon length (pb)/Tm (°C) | Efficiency (qPCR)* |
| --- | --- | --- | --- | --- | --- |
| GTACGCCGTGTTGTGGTGTG/ | BdSBPase | PCR | 0.2 | 330/62.1 | N/A |
| ACCGTCAAGGGGATCGAACG | Sequence |  |  |  |  |
| GTC TGC ACC ATC GTC AAC C | Bar | PCR | 0.3 | 444/57.0 | N/A |
| GAA GTC CAG CTG CCA GAA AC | Sequence |  |  |  |  |
| CTTTCGTAGTGGCCCTCAAG/ | BdSBPase | qPCR | 0.2 | 109/58.1 | 1.93 |
| ATCTTCCCTTCTCCGATGGT | Sequence |  |  |  |  |
| CCTCGTTCTACGGCGAGTC/ | TaSBPase | qPCR | 0.2 | 143/59.5 | 1.91 |
| TCGCCTTGGTCAGGAACT |  |  |  |  |  |
| GCTCTCCAACAACATTGCCAAC/ | Ta2291 | qPCR | 0.2 | 165/73.7 | 1.92 |
| GCTTCTGCCTGTCACATACGC |  |  |  |  |  |

* Primer efficiency as described by [Pfaffl (2001)](#_ENREF_32)
